# Does health technology assessment compromise access to pharmaceuticals?

**DOI:** 10.1007/s10198-023-01611-9

**Published:** 2023-06-19

**Authors:** Melanie Büssgen, Tom Stargardt

**Affiliations:** Hamburg Center for Health Economics, University of Hamburg, Hamburg, Germany

**Keywords:** AMNOG, Launch delay, Pharmaceuticals, HTA, Health policy, Price regulation, I18

## Abstract

In response to rapidly rising pharmaceutical costs, many countries have introduced health technology assessment (HTA) as a ‘fourth hurdle’. We evaluated the causal effect of HTA based regulation on access to pharmaceuticals by using the introduction of Germany’s HTA system (AMNOG) in 2011. We obtained launch data on pharmaceuticals for 30 European countries from the IQVIA (formerly IMS) database. Using difference-in-difference models, we estimated the effect of AMNOG on launch delay, the ranking order of launch delays and the availability of pharmaceuticals. We then compared the results for Germany to Austria, Czechia, Italy, Portugal and the UK. Across all six countries, launch delay decreased from the pre-AMNOG period (25.01 months) to the post-AMNOG period (14.34 months). However, the introduction of AMNOG consistently reduced the magnitude of the decrease in launch delay in Germany compared to the comparator countries (staggered DiD: + 4.31 months, *p* = 0.05). Our logit results indicate that the availability of pharmaceuticals in Germany increased as a result of AMNOG (staggered logit: + 5.78%, *p* = 0.009). We provide evidence on the trade-off between regulation and access. This can help policymakers make better informed decisions to strike the right balance between cost savings achieved through HTA based regulation and access to pharmaceuticals.

We feel honoured that our publication ‘Does health technology assessment compromise access to pharmaceuticals?’ is leading to discussions. In the following, we would like to respond to a comment made by Gandjour [3], which (1) relates to the fact that our results (unexpectedly) differ from those of a recently issued descriptive report by IQVIA, (2) argues that our findings may have been affected by a ‘catching-up effect’ and (3) in its last paragraph misinterprets our findings.

1. With respect to comparing our results to those of the recent IQVIA report [4], we believe that these simply lack comparability. In fact, the two analyses differ in nearly every methodological choice that would allow for a meaningful comparison. However, we believe that our methodological choices are, for the purpose of our analysis, superior to those in the IQVIA report in the following ways:
  - We define time-to-market as the difference between the ‘first international launch date (i.e. start of sales)’ and the ‘national launch date (i.e. start of sales)’, whereas the IQVIA report uses a different start date and a different end date, defining time-to-market as the period between ‘marketing authorization’ and the ‘point at which the product gains access to the reimbursement list’.
    We argue that the IQVIA report’s definition of start date (time of marketing authorization) is problematic for our purpose because it can be influenced by the management decisions of pharmaceutical companies. For example, a company may decide to apply for regulatory approval in countries in a specific order or with some time lags in between. This would then affect time-to-market as measured by IQVIA, whereas our measurement remains unaffected. Indeed, one might interpret IQVIA’s results for Macedonia (ranked 4th in time-to-market), Russia (ranked 6th in time-to-market) and Turkey (ranked 10th in time-to-market) as a consequence of its definition. All three countries experienced very few launches and, compared to the first international launch, a rather late filing for marketing authorisation. However, late market authorisation was followed by rapid inclusion in the national reimbursement list. Thus, while the IQVIA report shows Macedonia, Russia and Turkey to be top-performers with respect to time-to-market, this is, in our opinion, a result of IQVIA’s specific definition of the start date rather than reality.
    In contrast, our start date refers to the time when a pharmaceutical becomes available somewhere in the world for the first time. This could essentially be anywhere that a company has prioritised resources for studies or filing admission, etc. Thus, one could argue that IQVIA’s definition focuses on the administrative lag from reimbursement schemes (and only partially includes lags resulting from strategic behaviour to counter these), while our measure includes the full administrative lag (marketing authorisation, reimbursement) and also takes the strategic behaviour of companies fully into account.
  - The end date chosen in the IQVIA report for defining time-to-market also follows an approach different from ours. Whereas we link availability to the start of sales (and thus the point at which patients actually start to use the pharmaceutical), IQVIA links availability to an official reimbursement decision (surveyed from member companies of EFPIA).
    We are not convinced that the IQVIA report’s definition is more appropriate, because in each country’s reimbursement scheme, it is at least debatable which step constitutes an ‘official reimbursement decision’, particularly given that these data are elicited through surveys that might be subject to different perceptions of this definition across respondents and countries.
    For example, in Germany, a pharmaceutical is reimbursable the moment it launches. Then, there is a decision after 6 months about whether it has an added benefit beyond that of an appropriate comparator, and at month 12, the negotiations on reimbursement prices conclude. Companies may react to both of these steps by withdrawing a product from the market, which indeed they do in 8% of cases [5]. Which of these three events or dates would constitute the ‘official reimbursement decision’? In England, for our study period and as another example, NICE appraisals last for around 12 months and the NHS has another three months for implementation. In the meantime, each of the approx. 100 clinical commissioning groups (CCGs) could make the pharmaceutical available. IQVIA’s decision to use the timing of NICE decisions for England does not take account of the 3 months allowed for implementation, on the one hand, nor does it figure in earlier decisions by CCGs, on the other. Similar problems will arise in all regionalised health care systems with a mix of central decision-making and the right of regional authorities to overrule these decisions or to act earlier, such as those in Italy and Spain.
    This is why we favour our approach, which is based on hard sales data and follows the same definition in all countries.
  - The underlying samples differ. The IQVIA report limits itself to pharmaceuticals in oncology, orphan drugs and combination therapies, whereas we exclude combination therapies but include all other indication areas. In fact, pharmaceuticals in oncology and orphan drugs are the two market segments for which we would expect above average results for Germany. This is because (i) orphan drugs are granted added benefit in Germany per se and automatically reach the status of negotiations and (ii) cancer drugs have historically had much less trouble demonstrating improvement in patient-relevant outcomes and have generally outperformed all other indication areas with regard to the added benefit criterion of the Federal Joint Committee [1].
  - The time frames of the IQIVA report and ours differ. Whereas we look at all pharmaceuticals launched between 2003 and 2017, the IQVIA report focuses on launches between 2017 and 2021.
  - The follow-up of launches in the IQVIA report is limited to the date of its publication (July 2022), allowing only for a follow-up of about 1 year for all drugs launched in 2021 and for a follow-up of about 2 years for drugs launched in 2020. In contrast, our follow-up is between a minimum of 3 years for pharmaceuticals launched in 2017 and up to 17 years for pharmaceuticals launched in 2003. This will lead to very different results for time-to-market between the two publications because launch delays longer than one year (for 2021) or longer than two years (for 2020) simply cannot be observed in the IQVIA report.

In summary, it is not necessary from our point of view to reconcile our results with those of the IQVIA report, as suggested in the comment. It is a simple matter of fact that both publications have made very different choices with respect to the methods that were used. However, we are convinced—given the arguments presented above—that our choices are superior to those employed in the IQVIA report for the purpose of our research.

(B) With respect to the choice of comparators, the comment argues that our results may be affected by a ‘catching-up effect’—that is, the potential for reductions is higher for those countries with a longer initial time-to-market.

We discuss and acknowledge this in our limitations, but would like to point out that we also compared Germany (launch delay before AMNOG: 16.98/after AMNOG: 9.84) with the UK (launch delay before AMNOG: 18.74/after AMNOG: 9.59). The UK is the country that was ranked directly after Germany in launch delay before AMNOG and then switched its rank with Germany in the post-AMNOG period. Thus, according to the comment’s own reasoning, it is the best match that we could use. Nevertheless, we found an increase in launch delay of 2.0 months over time due to AMNOG when Germany was compared to the UK.

Moreover, when looking at the two EU countries with the earliest launch in Europe (the Netherlands and Sweden), which we did not use as comparators due to a lack of parallel pre-trends, we see that they reduced launch delay from 9.51 to 5.35 months in the case of the former and from 13.81 to 6.02 months in the case of the latter. Thus, the 9.84 months of average launch delay that we observed for Germany in the period after 2011 by no means represents the shortest launch delay possible.

Econometrically speaking, the countries to which we compared Germany all had a parallel trend in the pre-policy period. That is, the reductions in launch delay did not differ over time for any of the country comparisons to Germany before AMNOG (Büssgen and Stargardt [2] figures 1–6 and results from the placebo regressions). Thus, it is not apparent to us why the difference in ‘catching-up’ that we only start to observe after the introduction of AMNOG (and not before) should be interpreted as being unrelated to AMNOG.

(C) Lastly, the comment misinterprets and, in part, misquotes our conclusion. We do not dispute the general downward trend in launch delay over time across all countries (including Germany). In fact, we acknowledge it multiple times in our paper. Crucially, however, we observed differences-in-differences over time, providing evidence that this downward trend over time was weaker in Germany following the introduction of AMNOG compared to other countries. We did not argue, as misquoted by Gandjour [3], that ‘AMNOG (…) may lead to longer launch delays and thus later patient access in Germany’ in general but made very clear that this was relative to the development in other countries. Indeed, the last four words of the sentence in question, which—for whatever reason—are not quoted in the comment, are ‘compared to other countries’.

## Data Availability

The data upon which our analysis (IQVIA, formerly IMS Health) is based are proprietary and we used Stata SE 16 to conduct the analyses.
